# P144: A study of psycho-social behavior related to hand hygiene & co-relation with well-being of health care workers

**DOI:** 10.1186/2047-2994-2-S1-P144

**Published:** 2013-06-20

**Authors:** P Sharma, N Singh, N Taneja

**Affiliations:** 1 Medical Microbiology, Pgimer, India; 2 Psychology, D.A.V College, Chandigarh, India

## Introduction

Well-being at work is based on positive psychology,and is defined as a psychological state with positive affective links towards work (patient-care) and towards the organization (hospital). Well being of health care workers (HCWs) directly affects their relationship with patients. Hand hygiene (H.H) is the most effective measure for preventing cross-infection, so good compliance is highly desirable among HCWs.

## Objectives

To study psychosocial factors affecting H.H compliance & correlation between psycho-social behavior related to hand hygiene of HCW’s & their wellbeing.

## Methods

Study was conducted in 41 nurses. Self made questionnaire tested five main domains – H.H at home, In-Hospital H.H (elective & inherent), perceived peer group behavior, Attitudes & Non-compliance regarding H.H. Items were scored on 5 point and 7 point Lickert scales.WHO – 5 Well-being scale was used to measure well being. Spearman Correlation (r_s_) & percentages were used to interpret scores.

## Results

Hypothesis that H.H behavior at home (r_s_ =0.36), behavior in elective (r_s_=0.34) & inherent (r_s_=0.45) hospital H.H, perceived peer group behavior (r_s_= 0.32) & the attitudes (r_s_=0.35) will have a positive relationship with wellbeing whereas non-compliance will have a negative correlation (r_s_=-0.42) with wellbeing, was proved. HCW who washed hands regularly at home also showed good H.H compliance at work, 59% of the HCW admitted of not washing hands everytime, main reasons given for non-compliance were less time (50%), minor patient contact (51.2%) and work overload (52%). HCW had low awareness regarding frequently touched surfaces. Same peer group behaviour was more likely to increase compliance. Social desirability and actor-observer role were observed. Suggestions elicited from the participants to improve compliance to H.H included: banners in local language, use of electronic media to grab interest, proper availability of facilities like hand sanitizers, soaps, cold and warm water, proper placement of wash basins etc.

## Conclusion

Elective H.H is influenced by the attitudes which are learnt in the young ages. H.H is also important for well being of HCW. Though HCW were aware of the importance of H.H, their awareness about frequently touched surfaces was low.

## Disclosure of interest

None declared

